# Resistant anemia in a kidney transplant recipient: Pure red cell aplasia due to parvovirus B19 infection

**DOI:** 10.5339/qmj.2024.8

**Published:** 2024-02-10

**Authors:** Hassan Choudry, Fateh Chattah, Hilal Shalla, Farooq Mehr, Saddam Hussain Abbasi, Jorge Jesus-Silva

**Affiliations:** Department of Nephrology, University Hospitals of Leicester (UHL), Leicester, UK Email: hassanchoudry01@gmail.com ORCID iD: 0000-0001-6720-724X; Department of Respiratory Medicine, University Hospitals of Leicester (UHL), Leicester, UK

**Keywords:** Pure Red Cell Aplasia (PRCA), parvovirus B19 infection, kidney transplant, renal transplant, anemia, Intravenous Immunoglobulin (IVIG), tacrolimus, cyclosporine, mycophenolate mofetil, immunosuppression

## Abstract

Anemia in kidney transplant recipients can stem from a diverse array of etiologies, including dietary deficiencies, inflammatory processes, allograft dysfunction, as well as viral and bacterial infections. We present a case of refractory anemia in a 49-year-old male patient occurring within the initial month following a kidney transplant, which persisted despite numerous transfusions, posing a formidable challenge. The patient was maintained on the standard immunosuppressant regimen—Tacrolimus, Mycophenolate, and Prednisolone. Diagnostic evaluations eliminated well-established causes such as dietary deficiencies, gastrointestinal losses, and prevalent infections. Subsequently, after viral PCR testing, a diagnosis of Pure Red Cell Aplasia (PRCA) due to infection with parvovirus B19 was made. Although the patient had a reduction in the immunosuppression drugs and received a course of Intravenous Immunoglobulins (IVIG) on two separate occasions spanning two months, the anemia relapsed. Subsequently, after an additional dose of IVIG with further modification and reduction of the immunosuppressant regimen, including stopping the mycophenolate and switching tacrolimus with cyclosporine, the patient ultimately achieved successful resolution of his symptoms and a significant decrease in viral load. Our case highlights the significance of unconventional etiologies when confronted with anemia in the setting of kidney transplantation. Furthermore, it also provides further insights into therapeutic avenues for addressing PRCA in kidney transplant recipients.

## Introduction

Anemia in kidney transplant recipients is one of the leading causes of morbidity, with some studies highlighting its association with mortality as well.^1,2^ The multifactorial etiology ranges from iron deficiency, allograft dysfunction, immunosuppression, and chronic infections (including CMV, EBV, and Parvovirus B19).^3^

Parvovirus B19 is a non-enveloped, single-stranded DNA virus of the Parvoviridae family, which can affect both adults and children.^4^ The infection is usually self-limiting in individuals with a healthy immune system. However, it can cause the so-called fifth disease, classic childhood rashes comprising arthralgias, fever, malaise, and hydrops fetalis in pregnant women.^5^ In immunocompromised people, it causes Pure Red Cell Aplasia (PRCA), a disease resulting from viral bone marrow infection. Furthermore, in kidney transplant recipients, it has also been shown to have associations with acute and chronic allograft dysfunction, antibody-mediated rejection, collapsing glomerulopathy, and thrombotic microangiopathy.^6-8^

The mechanism of Parvovirus-related anemia involves the suppression of marrow erythrogenic precursor cells. Parvovirus B19 binds to P-antigen on erythroid cells, entering and replicating in these cells and eventually causing lysis.^5^ The resultant anemia, albeit very mild and asymptomatic in normal populations, becomes severe when superimposed upon chronic anemic conditions such as kidney disease, patients on immunosuppressant medications, or those with Thalassemia and Sickle Cell Disease.

In kidney transplant recipients (KTRs) who have anemia post-transplant, the prevalence of PRCA due to B19 is between 7% and 12%.^9,10^ The anemia usually appears within the first month of infection post-transplant and is noticed by symptoms or routine laboratory testing.^11^ It is a normocytic and normochromic anemia characterized by decreased reticulocyte count and decreased erythrocytic blast cells in the bone marrow. Non-PRCA causes are usually investigated first, and the exclusion of these prompts clinicians to test for viral etiology.^12^ In a small number of cases, however, diagnosis is made by determining the hallmark features of PRCA on bone marrow biopsy.

No specific therapy exists for PRCA in the context of kidney transplants; the anemia is unresponsive to erythropoietin but usually responds to a decrease in conventional immunosuppression.^13^ Common immunosuppressants used in KTRs are Mycophenolate and Tacrolimus, which work by different pathways, inhibiting lymphocyte proliferation and activation, respectively; these cells are the key agents in the control of viral infections, and their impairment increases the risk of infections. Reducing the dose of mycophenolate is the first strategy in patients with viral infections, followed by a reduction in calcineurin-inhibitor trough levels (tacrolimus and cyclosporine). Intravenous immunoglobulin is used in most protocols, although there have been no controlled trials, and its dosing varies by the center.^14^

We report a case of a patient developing anemia within a month after his kidney transplant. Following comprehensive diagnostic assessments, the patient was identified as having Pure Red Cell Aplasia (PRCA), secondary to Parvovirus B19 infection. The condition posed a notable challenge in terms of management because of the transplant, persisting for several months before eventually responding to therapeutic interventions.

## Case Report

A 49-year-old male patient was hospitalized due to symptomatic anemia from our clinic on July 21^st^, 2022. The patient had undergone a right-sided cadaveric kidney transplant three weeks prior, donated after circulatory death (DCD) on July 2^nd^, 2022, in our center on the right side. Pre-transplant serology showed that the patient was positive for both Cytomegalovirus (CMV) and Epstein Barr Virus (EBV) PCR; serology for parvovirus B19 is not routinely done in our patients, so his status was unknown. He was given induction immunosuppression with Basiliximab and intravenous methylprednisolone and was started on a maintenance regimen comprising tacrolimus, mycophenolate, and prednisolone (on a tapering dose) as per hospital guidelines. The patient was also started on *Pneumocystis jerovici* prophylaxis with Trimethoprim/sulfamethoxazole. He attended a regular transplant clinic three times per week, with significant improvement in his creatinine, urea, and other toxins, but with ongoing reductions in his hemoglobin, reaching 6.6 mg/dL by the 3^rd^ week post-transplant; this was normocytic and normochromic, with low reticulocytes and normal serum iron stores, B12, and folate.

He was admitted with no symptoms or signs of active bleeding and an unremarkable physical examination; he was transfused with five units over five days and underwent an upper endoscopy, which revealed hiatus hernia, oesophagitis, with multiple ulcers, and erosive duodenitis. The endoscopic findings were deemed to be a source of the patient’s anemia, and he was treated with Intravenous Omeprazole. His anemia improved after transfusions and inpatient management, and he was discharged after four days with a plan for a repeat endoscopy eight weeks later.

A month and a half post-transplant, he was admitted again due to symptomatic anemia (Hb: 6.3 mg/dL). Considering his previous endoscopy results, the patient had a repeat endoscopy, which revealed erosive duodenitis without any associated bleeding. He was started on a *Helicobacter Pylori* eradication regimen and high-dose antiulcer medications (Omeprazole, Metronidazole, and Amoxicillin/clavulanate). The patient received five units of RBC transfusion over four days and was discharged with Darbopoetin Alfa.

The refractory anemia raised suspicion of secondary causes of anemia during the same admission. A virology screen was carried out, including HBV, HCV, Parvovirus B19, CMV, EBV, and BK Virus; Parvovirus B19 DNA PCR came back positive at 78.7 billion parvovirus DNA IU/mL (1 parvovirus IU is equivalent to 0.65 DNA copies). The patient’s immunosuppressant regimen was adjusted by reducing the dose of mycophenolic acid and ultimately switching it for azathioprine and titrating his tacrolimus trough levels (from 8-10 to 6-8 ng/mL). He also received intravenous immunoglobulin (IVIG) 20 grams over three days; he was discharged with his blood hemoglobin at 9.0 mg/dL and P-B19 viral load of 21.2 million IU/mL.

Despite the improvement, he was readmitted for a third time for worsening anemia about three months post-transplant (Hb: 6.4mg/dL). Parvovirus B19 PCR revealed > 100 billion DNA IU/mL (greater than the upper detection limit of the test) on two occasions; he was given a second session of 20g IVIG for four days. Tacrolimus was further decreased (trough level 3-5 ng/mL), and azathioprine was stopped. He was also transfused with five units of blood during this admission and was discharged on Day 7 after completing the IVIG course. Hemoglobin at discharge was 8.0 mg/dL.

The patient had a brief admission during the fourth-month post-transplant with anemia (Hb: 6.9 mg/dL), and he was transfused with two units of blood. His parvovirus B19 load remained >100 billion IU/mL. His case was discussed in a multidisciplinary meeting with infectious diseases, immunology, and transplant, and IVIG was considered futile for this admission. However, it was decided to reduce his immunosuppresion even more, and hence his tacrolimus dose was reduced.

During his fifth month post-transplant, the patient was admitted from the clinic with anemia. His hemoglobin this time was 7.4 mg/dL, and he required four RBC transfusions and a third session of IVIG infusion (20 grams in total); tacrolimus was switched to cyclosporin to keep a CNI in his regimen, although less potent. Following that intervention, his Parvovirus B19 viral load reduced dramatically: 11.9 million DNA IU/mL and then 59,400 DNA IU/mL. His hemoglobin also presented a significant increase by the end of November (five months post-transplant); it was the first time he achieved Hb >10 mg/dL (even with transfusions, his highest had been 9.0). His transplant and hemoglobin remained stable until June 2023; at the beginning of the month, his GFR was 40, and his Hb was 12.8, with cyclosporine (trough level ~150) and prednisolone 5 mg OD. Unfortunately, later that month, he presented an aggressive episode of antibody-mediated rejection; upon admission, his GFR was 14, Hb 9.6 mg/dL, and Parvovirus viral load 690 IU/mL.

## Discussion

We describe an archetypal Pure Red Cell Aplasia (PRCA) case due to parvovirus B19 in a kidney transplant recipient. The onset, diagnostic approach, and clinical progression are comparable to what has frequently been documented in the literature.^15^ However, the response to the treatment has varied.

The most appropriate treatment option for PRCA in renal transplant patients is Intravenous Immunoglobulins (IVIG), which replenishes the patient’s serum with missing gamma globulins.^14^ Significant side effects include transfusion reactions and the development of anti-HLA antibodies in the long term. Our patient developed fever and chills upon the first infusion, which were controlled by pretreatment with steroids and antihistamines upon subsequent infusions. The response rate to IVIGs is debatable in the literature, but most patients need one session.^12,15^ Our case, however, required three IVIG treatment sessions over four months as he relapsed after the first two treatment sessions.

**Figure 1.**
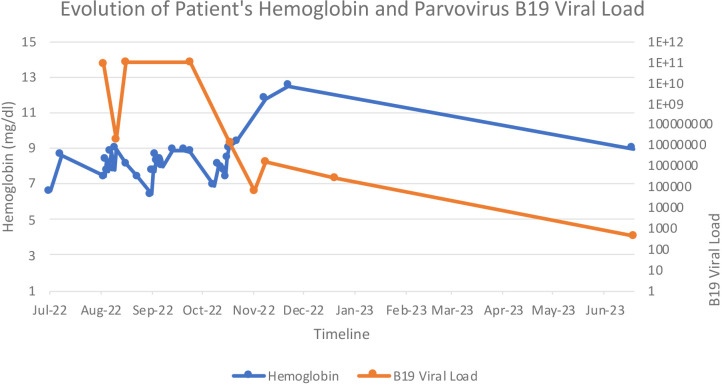
The line graph shows the patient’s hemoglobin change and parvovirus B19 load.

Mycophenolate is known to inhibit both B and T lymphocytes and, in turn, antibody production; hence, its effect on immunity is more pervasive than that of the other two drugs.^16^ It is, therefore, recommended to stop mycophenolate entirely and start the patient on a lower target for tacrolimus.^17,18^ Another recommendation is to replace tacrolimus with cyclosporine.^18^ We opted for both options for our patient (Figure 1). When the first IVIG treatment failed to bring sustained remission, his regimen was changed to cyclosporine, which was likely pivotal in controlling his infection. However, it is hard to determine which of these two management modalities contributed more toward resolving the disease.

Altheaby et al. (2021) mentioned in a case series that all three of their cases had to change their immunosuppression regimen and stop mycophenolate, while two of their patients received IVIGs, the long-term resolution of viremia was only achieved after switching from mycophenolate, a strategy closely mimicking our case.^17^ Allograft dysfunction has been described with the ParvoB19 infection in patients.^6,19^ However, our patient’s graft function largely remained normal throughout his treatment and admissions. We believe that the episode of rejection he presented within the first year of transplantation is a consequence of the reduced potency of his immunosuppression regimen, highlighting the difficult situation that transplant patients present when it is desired to keep rejection at bay but not at the cost of life-threatening infections.

## Conclusion

Parvovirus B19 infection in renal transplant recipients remains a threat and requires clinical vigilance in diagnosis and management. PRCA must be excluded in any case of anemia, and appropriate steps must be taken for management as early as possible.

## Declarations

### Ethics approval and consent to participate

Consent was gained from the patient verbally. Ethical approval was not required for this case report.

### Availability of data and materials

Data sharing is not applicable.

### Competing interests

The authors declare that they have no competing interests.
